# Design, synthesis, and biological evaluation of novel androst-17β-amide structurally related compounds as dual 5α-reductase inhibitors and androgen receptor antagonists

**DOI:** 10.1080/14756366.2019.1654469

**Published:** 2019-08-30

**Authors:** Kejing Lao, Guoliang Xun, Xingchun Gou, Hua Xiang

**Affiliations:** a Institute of Basic and Translational Medicine, Shaanxi Key Laboratory of Brain Disorders, Xi’an Medical University, Xi'an, PR China;; b Jiangsu Key Laboratory of Drug Design and Optimization, China Pharmaceutical University, Nanjing, PR China;; c Department of Medicinal Chemistry, School of Pharmacy, China Pharmaceutical University, Nanjing, PR China;; d Abbisko Therapeutics Co Ltd, Shanghai, PR China

**Keywords:** 5α-reductase Inhibitors, androgen receptor antagonists, prostate cancer

## Abstract

Prostate cancer (PCa) is the second leading cause of death in men. Apart from androgen receptor, 5α-reductase has also been recognized as a potential target. In this study, a series of androst-17β-amide compounds have been designed and synthesized targeting both AR and 5α-reductase. Their anti-proliferation activities were evaluated in AR + cell line 22RV1 and AR − cell line PC-3. The results indicated that most of the synthesized compounds inhibited the testosterone-stimulated cell proliferation with good selectivity and safety. Among all the compounds, androst[3,2-c]pyrazole derivatives **(9a–9d)** displayed the best inhibition activity comparable with flutamide. Moreover, most of the synthesized compounds displayed good 5α-reductase inhibitory activities with IC_50_ lower than 1 μM. The docking result of **9d**-AR indicated that AR was forced to expands its binding cavity and maintain an antagonistic conformation since the steric hindrance of **9d** impeded H12 transposition. Overall, compound **9d** can be identified as a potential dual 5α-reductase inhibitor and AR antagonist, which might be of therapeutic importance for PCa treatment.

## 1. Introduction

Prostate cancer (PCa) is the second leading cause of death in men only less than lung cancer. It was estimated that around 220,800 cases were diagnosed in the United States in 2015 alone. In China, that number was 60,300 and has increased rapidly over the last 10 years^1^. Androgens, including testosterone (T) and dihydrotestosterone (DHT), and androgen receptor (AR) signalling pathway are essential for prostate development and homeostasis^2^. Huggins et al.^3^ introduced androgen deprivation therapy (ADT) for advanced and metastatic PCa in 1941. Thereafter, androgen ablation therapy has been shown to produce the most beneficial responses in multiple settings in PCA patients.

**Figure 1.**
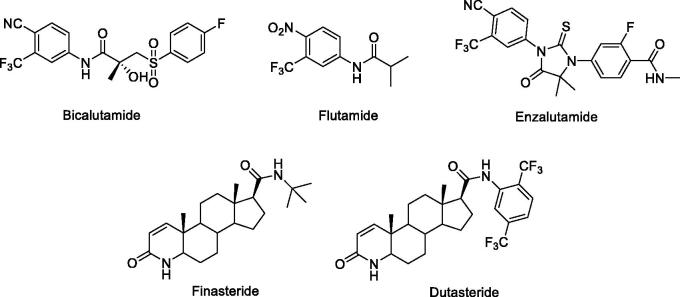
The AR antagonists and 5α-reductase inhibitors in clinical.

As the substantial clinical efficacy with AR blockade in PCa patients, AR has been recognized as an attractive target for the treatment of PCa. A number of small molecular AR antagonists, such as bicalutamide and flutamide (Figure 1) have been shown good therapeutic effects in clinic^4^. Newly approved enzalutamide (Figure 1) is a new generation AR antagonist which is effective for bicalutamide-resistant tumours and has been used in CRPC patients^5^. Although AR antagonists shown great benefits in treating PCa, drug resistances caused by AR mutation occur spontaneously in PCa^6^^,^^7^ as well as altered steroidogenesis^8^ underlies the emergence of castration-resistant prostate cancer (CRPC) within 2–3 years after starting ADT. Therefore, the exploration of developing new anti-PCa agents with increased activity and fewer side effects is still in urgent.

Recently, it has been shown that the low levels of DHT in the prostate after ADT is a result of intratumoral androgen synthesis^9^. 5α-reductases, which are NADPH-dependent enzymes, are responsible for the reduction of T to DHT. The 5α-reductase family is composed of three isozymes, with the types 1 and 2 being the most known. Type 1 5α-reductase (5αR-1) is mainly expressed in prostate epithelial cells as well as in skin and liver at an optimal pH range of 6.0–8.5, while the type 2 5α-reductase (5αR-2) at an optimal pH 5.5, is mainly found in prostatestromal compartment and other genital tissues^10^. More recently, type 3 isozyme was identified in castration-resistant PCa cells as well as in other tissues such as the pancreas, brain, skin and adipose tissues^11^. The 5α-reductase levels, particularly type 1, appear to increase during the disease course of prostatic intraepithelial neoplasia and PCa, with greater expression occurring as the disease progresses^12^. Therefore, the inhibition of 5α-reductase could potentially reduce the risk of PCa development, prevent disease progression, and treat existing disease.

5α-reductase inhibitors, like finasteride and dutasteride (Figure 1), were used in the clinic for the treatment of BPH and were also proposed for chemoprevention and treatment of PCa. Dutasteride blocks both type 1and type 2 isoenzymes and has an inhibitory effect in PCa^13^^,^^14^. It is currently being studied as a chemopreventive agent and in combination with other androgen-reducing agents in PCa treatment. It was found that combination of dutasteride and enzalutamide synergistically inhibited tumour cell proliferation^15^. In our previous study, several androstene-17β-carboxamides has been reported as dual 5α-reductase inhibitors and antiandrogens showing good anti-proliferative activities in a human PCa cell line LNCaP cells and PC-3 cells^16^. These findings supported clinical studies with combinations of a 5α-reductase inhibitor and antiandrogens for the first line treatment of PCa and CRPC.

**Figure 2.**
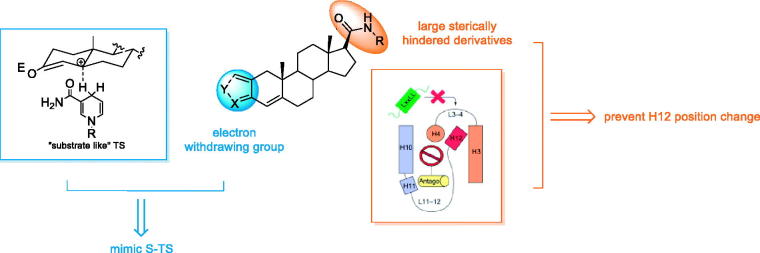
The rational design strategy of dual AR/5α-reductase inhibitors.

Considerable studies have been focused on the modifications of substitutions at C-17 position of steroid skeleton. Among them, aniline moiety has been widely used as functional groups in compounds targeting androgen axis^16^^,^^17^. This bulk in antagonist drugs, though small, is thought to be essential in pushing AR helix12 (H12) away from the binding pocket, disturbing the agonist conformation of the receptor^18^ (Figure 2).

As the 5α-reductase isozymes have not been purified and crystallized due to their unstable nature, the design and optimisation of novel 5α-reductase inhibitor mainly relies on structure-based optimisation approach. 5α-reductase inhibitors are possible to postulate two different transition states (TS): the “substrate like” TS (S-TS) and the “product like” TS (P-TS) (Figure 2)^19^. For S-TS inhibitors, stabilisation of the proposed enolate intermediate is thus an obvious strategy for the design of potential inhibitors, and a number of compounds that satisfy this requirement have been produced. Thus it can be seen that introducing electron-withdrawing group in A ring and B ring is beneficial for inhibitory activity.

Thus, we set out to design and synthesis a number of novel dual 5α-reductase and AR antagonists by involving electron-withdrawing group at A ring of androst-17β-amides (Figure 2). The anti-proliferative activities of synthesized compounds were evaluated against 2 different PCa cell lines. Their inhibitory activity 5α-reductase isozyme types 1 and 2 were further assessed, respectively.

## 2. Materials and methods

### 2.1. Chemistry

Melting points of compounds were measured on a RY-1 melting point apparatus and were uncorrected. Nuclear magnetic resonance (^1^H NMR) spectra were recorded on a Bruker AV-300 (300 MHz) spectrometer as deuterochloroform (CDCl_3_) solutions using tetramethylsilane (TMS) as an internal standard (δ = 0) unless noted otherwise. Electron impact mass spectral (EI-MS) data were obtained on a SHIMADZU GCMS-QP2010 system. All chemicals were purchased from commercial sources and were used without further purification unless otherwise noted. The solvents (such as MeOH, EtOAc, EtOH, CH_2_Cl_2_, and others) were C.P. grade purchased from Nanjing Chemical Co., Ltd. and used without further purification. Column chromatography (CC) was carried out on silica gel (200–300 mesh, Qingdao Ocean Chemical Company, China). Thin-layer chromatography (TLC) analyses were carried out on silica gel GF254 (Qingdao Ocean Chemical Company, China) glass plates (2.5 × 10 cm with 250 µm layer). Concentration and evaporation of the solvent after reaction or extraction was carried out on a rotary evaporator operated at reduced pressure.

#### 2.1.1. Synthesis of 3β-hydroxyl-5-pregnen-20-one-3-acetate(2)

To the solution of 3β-hydroxyl-5,16-Pregnadien-20-one-3-acetate (6 g, 16.8 mmol) in 180 ml EtOAc, 0.6 g Raney Ni was slowly added at 0 °C. The reaction mixture was stirred at 25 °C for 1 h. After filtration, the filtrate was evaporated to provide white solid 5.924 g, yield 98.18%. ESI-MS *m/z*: 381[M + Na]^+^.

#### 2.1.2. Synthesis of 3β-hydroxyl-5-pregnen-20-one(3)

To the solution of **2** (5.92 g, 16.5 mmol) in 36 ml MeOH, 9 ml 20% K_2_CO_3_ aqueous solution was added. The reaction mixture was refluxed for 2 h. After cooled to room temperature, the resulting precipitate was filtered, washed with water, and dried to give white solid 4.85 g, yield 92.81%. ESI-MS *m/z*: 339[M + Na]^+^.

#### 2.1.3. Synthesis of 3-oxo-4-pregnen-20-one(4)

To the solution of **3** (7 g, 22 mmol) in 112 ml totuene, cyclohexanone (21 ml, 0.2 mol) was added. After circumfluence to repel water for 2 h, aluminiumisopropoxide (1.05 g, 5.1 mmol) was added. After refluxed for another 1.5 g, the resulting mixture was poured into 10 ml cold 10% NaOH aqueous solution. The solvent was evaporated by steam distillation to provide yellow solid 6.3 g, yield 90.03%

#### 2.1.4. Synthesis of 17β-carboxy-androst-4-ene-3-one(5)

To the solution of NaOH (5.9 g, 143 mmol) in 50 ml water, Br_2_ (1.9 ml, 37 mmol) was slowly added at 0 °C to provide NaOBr aqueous solution. Resolving **4** (3.5 g, 11 mmol) in 105 dioxane and 36 ml water, the NaOBr aqueous solution was added. After stirred at room temperature for 1.5 h, 19 ml 10% Na_2_SO_3_ was added and refluxed for 15 min. After cooled to room temperature, 2 N hydrochloric acid was added to adjust PH to 2 in ice bath. Dioxane was evaporated and the resulting precipitate was filtered, washed with water, and dried to give crude product. Recrystallized by acetone to provide white solid 2.6 g, yield 75.48%.

#### 2.1.5. General procedure for the preparation of 2-hydroxymethylene-17β-formamido-androst- 4-ene-3-one (7a–7e)

To a solution of **5** (1 g, 3.16 mmol), anhydrous pyridine (0.32 ml, 4.12 mmol) in 15 ml anhydrous toluene, oxalyl chloride (0.34 ml, 3.56 mmol) in 7 ml anhydrous toluene was added drop wise at 0 °C. The resulting solution was stirred at room temperature 1.5 h then 7.9 mmol corresponding amine was added. The mixture was stirred at room temperature for another 8 h. 30 ml water was added and the reaction mixture was extracted three times with ethyl acetate. It was dried with sodium sulfate and the solvent was removed in vacuum. This compound was used in the next step without purification.

To the solution of 1.23 mmol corresponding amido product in 6 ml THF, NH (0.3 g, 7.4 mmol) was added. The mixture was stirred at room temperature for 0.5 h. 4 ml ethyl formate was added and the reaction mixture was refluxed for 2 h. After cooled to room temperature, the reaction mixture was poured into 20 ml water. Adjust PH to 2 by 2 N hydrochloric acid then extracted three times with ethyl acetate. It was dried with sodium sulfate and the solvent was removed in vacuum. The compound was purified by column chromatography (PE/Ethyl acetate, 2:1).

#### 2.1.6. General procedure for the preparation of 2-hydroxymethylene-17β-(N-isobutyl-carboxamide)-androst-4-ene-3-one (7a)

Yield: 55% of pure product, m.p(0).152–158 °C. ^1^H NMR (CDCl_3_, 300 Hz) δ: 0.8 (s, 3H, 18-CH_3_), 1.08 (s, 3H, 19-CH_3_), 1.47 (s, 9H, t-Bu), 5.83 (s, 1H, 4-H), 7.30 (s, 1H, 2-ethylene), 13.82 (s, 1H, –OH) ppm. 13C NMR (CDCl_3_, 300 Hz) δ: 13.30, 17.58, 20.89, 24.22, 25.34, 26.84, 30.95, 31.96, 35.30, 37.01, 38.09, 42.7, 46.62, 50.16, 52.66, 55.35, 102.94, 109.85, 122.50, 123.96, 164.69, 169.55 ppm. HRMS (ESI): *m/z* [M + H]^+^. Calcd for C_25_H_37_NO_3_: 399.2773; Found: 399.2778.

#### 2.1.7. General procedure for the preparation of 2-hydroxymethylene-17β-(piperidine-1-carbonyl)-androst-4-ene-3-one(7b)

Yield: 42% of pure product, m.p. 105–110 °C. ^1^H NMR (CDCl_3_, 300 Hz) δ: 0.82 (s, 3H, 18-CH_3_), 1.04 (s, 3H, 19-CH_3_), 3.2–3.0 (m, 4H, CH_2_-N-CH_2_), 5.76 (s, 1H, 4-H), 13.80 (s, 1H, −OH) ppm. 13C NMR (CDCl_3_, 300 Hz) δ: 12.98, 13.36, 14.18, 17.51, 20.8, 24.21, 25.25, 29.16, 30.9, 31.92, 35.2, 36.99, 38.22, 39.36, 39.75, 41.39, 44.39, 50.38, 52.58, 55.24, 105.9, 122.41, 164.62, 169.65, 171.69 ppm. HRMS (ESI): *m/z* [M + H]^+^. Calcd for C_26_H_37_NO_3_: 411.2777; Found: 411.2773.

#### 2.1.8. General procedure for the preparation of 2-hydroxymethylene-17β- (N,N-diethy-carboxamide)-androst-4-ene-3-one(7c)

Yield: 68% of pure product, m.p. 153–159 °C.^1^H NMR (CDCl_3_, 300 Hz) δ: 0.84 (s, 3H, 18-CH_3_), 1.03 (s, 3H, 19-CH_3_), 3.2 8–3.02 (m, 4H, CH_2_–N–CH_2_), 5.37 (s, 1H, –CONH–), 5.83 (s, 1H, 4-H), 7.30 (s, 1H, 2-ethylene), 13.78 (s, 1H, –OH) ppm. ^13^C NMR (CDCl_3_, 300 Hz) δ: 12.68, 19.72, 20.83, 23.15, 23.97, 28.12, 29.20, 30.83, 31.91, 35.22, 37.07, 37.92, 39.40, 43.14, 46.34, 52.60, 55.04, 56.69, 105.92, 122.53, 152.91, 156.49, 164.72, 173.14 ppm. EI-MS *m/z*: 387 (M^+^); HRMS (ESI): *m/z* [M + H]^+^. Calcd for C_25_H_37_NO_3_: 399.2779; Found: 399.2773.

#### 2.1.9. General procedure for the preparation of 2-hydroxymethylene-17β-(N-(3,4-dimethoxyphenethyl)-carboxamide)-androst-4-ene-3-one(7d)

Yield: 65% of pure product, m.p. 121–129 °C. ^1^H NMR (CDCl_3_, 300 Hz) δ: 0.74 (s, 3H, 18-CH_3_), 1.01 (s, 3H, 19-CH_3_), 3.53 (m, 4H-, N–CH_2_–CH_2_–Ar), 3.92 (s, 6H, –OCH_3_), 5.35 (s, 1H, –CONH–), 5.89 (s, 1H, 4-H), 6.00 (s, 1H, Ar-2H), 6.79 (d, *J* = 6.70, 1H, Ar-5H), 6.88 (d, J = 6.87, 1H, Ar-6H) ppm. 13C NMR (101 MHz, CDCl_3_) δ: 13.07, 13.24, 18.04, 21.25, 23.57, 24.44, 31.33, 32.38, 35.44, 35.73, 37.57, 38.2, 39.88, 40.56, 43.57, 53.1, 55.5, 55.87, 55.97, 56.91, 57.03, 76.71, 77.03, 77.35, 106.38, 111.41, 111.94, 120.66, 123, 131.44, 147.75, 149.13, 165.39, 169.76, 172.47, 188.95 ppm. HRMS (ESI): *m/z* [M + H]^+^. Calcd for C_31_H_41_NO_5_: 507.2990; Found: 507.2985.

#### 2.1.10. General procedure for the preparation of 2-hydroxymethylene-17β-(N-cyclopropylamino-carbonyl)-androst-4-ene-3-one (7e)

Yield: 73% of pure product, m.p. 125–130 °C. ^1^H NMR (CDCl_3_, 300 Hz) δ: 0.72 (s, 3H, 18-CH_3_), 1.09 (s, 3H, 19-CH_3_), 2.76 (m, 1H, –NH-CH–), 5.82 (s, 1H, 4-H), 7.41 (s, 1H, 2-ethylene), 13.79 (s, 1H, –OH) ppm. 13C NMR (CDCl_3_, 300 Hz) δ: 5.85, 6.30, 6.46, 12.66, 17.55, 20.87, 22.04, 22.49, 23.01, 23.95, 30.82, 31.89, 35.19, 37.04, 37.87, 39.39, 43.2, 52.58, 55.02, 55.70, 56.20, 105.9, 122.50, 164.75, 169.43, 179.44, 188.59 ppm. HRMS (ESI): *m/z* [M + H]^+^. Calcd for C_24_H_33_NO_3_: 383.2459; Found: 383.2460.

#### 2.1.11. General procedure for the preparation of 2-methylene-17-formamido-androst-4-ene-3-one (8a–8e)

To the solution of 1 mmol corresponding 2-hydroxymethylene product in 8 ml acetone, 0.33 ml formaldehyde was added. The resulting mixture was stirred at room temperature for 2 h. Upon completion, the reaction mixture was poured into 30 ml water and extracted with ethyl acetate. The combined organic layers were dried and the solvent was evaporated *in vacuo* to provide yellow solid. The compound was purified by column chromatography (PE/ethyl acetate, 2:1).

#### 2.1.12. General procedure for the preparation of 2-methylene-17β-(N-isobutyl-carboxamide)-androst-4-ene-3-one (8a)

Yield: 55% of pure product, m.p. 168–175 °C. ^1^H NMR (CDCl_3_, 300 Hz) δ: 0.75 (s, 3H, 18-CH_3_), 1.07 (s, 3H, 19-CH_3_), 1.47 (d, 6H, CH-(CH_3_)_2_), 3.52 (m, 2H, –NH–CH_2_–), 5.20 (s, 1H, 2-ethylene), 5.81 (s, 1H, 4-H), 5.91 (s, 1H, 2-ethylene) ppm. 13C NMR (75 MHz, CDCl_3_) δ: 13.00, 13.40, 14.22, 18.04, 20.69, 24.19, 25.30, 31.34, 32.20, 35.12, 38.22, 39.75, 40.28, 41.39, 44.39, 44.52, 50.41, 52.61, 55.32, 76.17, 76.59, 77.02, 119.75, 123.95, 140.79, 171.45, 171.65, 188.33 ppm. HRMS (ESI): *m/z* [M + H]^+^. Calcd for C_25_H_37_NO_2_: 383.2824; Found: 383.2897.

#### 2.1.13. General procedure for the preparation of 2-methylene-17β-(piperidine-1-carbonyl)-androst-4-ene-3-one (8 b)

Yield: 42% of pure product, m.p. 150–155 °C. ^1^H NMR (CDCl_3_, 300 Hz) δ: 0.67 (s, 3H, 18-CH_3_), 1.00 (s, 3H, 19-CH_3_), 2.9 6–3.55 (m, 4H, –NH–(CH_2_)_2_–), 5.09 (s, 1H, 2-ethylene), 5.71 (s, 1H, 4-H), 5.81 (s, 1H, 2-ethylene) ppm. 13C NMR (75 MHz, CDCl_3_) δ: 13.49, 13.9, 14.71, 18.54, 21.22, 24.7, 25.84, 31.88, 32.7, 35.66, 38.77, 40.27, 40.79, 41.91, 44.9, 45.06, 50.97, 53.17, 55.88, 76.61, 77.03, 77.46, 120.21, 124.48, 141.31, 171.86, 172.18, 188.81 ppm. HRMS (ESI): *m/z* [M + H]^+^. Calcd for C_26_H_37_NO_2_: 395.2832; Found: 395.2824.

#### 2.1.14. 2-methylene-17β-(N,N-diethy-carboxamide)-androst-4-ene-3-one (8c)

Yield: 68% of pure product, m.p. 98–105 °C. ^1^H NMR (CDCl_3_, 300 Hz) δ: 0.61 (s, 3H, 18-CH_3_), 1.12 (s, 3H, 19-CH_3_), 2.8 7–3.09 (m, 4H, –NH– (CH_2_)_2_–), 5.09 (s, 1H, –CONH–), 5.30 (s, 1H, 2-ethylene), 5.70 (s, 1H, 4-H),5.80 (s, 1H, 2-ethylene) ppm. ^13^C NMR (75 MHz, CDCl_3_) δ: 13.49, 13.90, 14.71, 18.54, 21.22, 24.70, 25.84, 31.88, 32.7, 35.66, 38.77, 40.27, 40.79, 41.91, 44.9, 45.06, 50.97, 53.17, 55.88, 76.61, 77.03, 77.46, 120.21, 124.48, 141.31, 171.86, 172.18, 188.81 ppm. HRMS (ESI): *m/z* [M + H]^+^. Calcd for C_25_H_37_NO_2_: 283.2826; Found: 283.2824.

#### 2.1.15. General procedure for the preparation of 2-methylene-17β-(N-(3,4-dimethoxyphenethyl)-carboxamide)-androst-4-ene-3-one (8d)

Yield: 65% of pure product, m.p. 74–78 °C. ^1^H NMR (CDCl_3_, 300 Hz) δ: 0.74 (s, 3H, 18-CH_3_), 1.01 (s, 3H, 19-CH_3_), 3.53 (m, 4H, N-CH_2_-CH_2_-Ar), 5.29 (s, 1H, 2-ethylene), 5.35 (s, 1H, –CONH–), 3.92 (s, 6H, -OCH_3_), 5.89 (s, 1H, 4-H), 6.00 (s, 1H, 2-ethylene), 6.85 (m, 3H, Ar-H) ppm. 13C NMR (75 MHz, CDCl_3_) δ: 12.59, 17.54, 20.72, 23.04, 23.93, 30.81, 31.88, 34.94, 35.18, 37.03, 37.63, 39.36, 40.09, 43.07, 52.55, 54.95, 55.34, 55.43, 56.47, 76.18, 76.6, 77.03, 110.77, 111.33, 120.14, 122.49, 130.91, 147.14, 148.52, 164.75, 169.43, 172.02, 188.58 ppm. HRMS (ESI): *m/z* [M + H]^+^. Calcd for C_31_H_41_NO_4_: 491.3036; Found: 491.3107.

#### 2.1.16. General procedure for the preparation of 2-methylene-17β-(N-cyclopropylamino-carbonyl)-androst-4-ene-3-one (8e)

Yield: 73% of pure product, m.p. 135–142 °C. ^1^H NMR (CDCl_3_, 300 Hz) δ: 0.53 (m, 4H, –CH_2_–CH_2_–), 0.69 (s, 3H, 18-CH_3_), 1.06 (s, 3H, 19-CH_3_), 2.75 (m, 4H, –NH–CH–), 5.45 (s, 1H, 4-H), 6.23 (s, 2H, 2-ethylene) ppm. 13C NMR (101 MHz, CDCl_3_) δ: 13.18, 18.56, 20.20, 21.20, 23.68, 24.42, 28.61, 29.69, 31.75, 32.65, 35.63, 38.39, 40.77, 43.64, 45.07, 46.86, 53.11, 55.59, 57.19, 76.70, 77.01, 77.33, 120.25, 124.54, 141.28, 171.70, 172.39, 188.77 ppm. HRMS (ESI): *m/z* [M + H]^+^. Calcd for C_24_H_33_NO_2_: 367.2520; Found: 367.2511.

#### 2.1.17. General procedure for the preparation of General procedure for the preparation of 4-oxa-17-formamido-5α-androst-3-one (9a–9e)

To the solution of 1 mmol, corresponding 2-hydroxymethylene product in 8 ml hydrazine hydrate. The resulting mixture was refluxed for 2 h. Upon completion, the reaction mixture was poured into 30 ml water. The resulting precipitate was filtered, washed with water, and dried to give white solid. The compound was purified by column chromatography (PE/Ethyl acetate, 1:1).

#### 2.1.18. General procedure for the preparation of 17β-(N-isobutyl-carboxamide)-androst[3,2-c]pyrazole-4-ene (9a)

Yield: 20.01% of pure product, m.p.:189–195 °C.^1^H NMR(CDCl_3_, 300 MHz) δ: 0.51 (m, 2H, −CHCH_2_CH_2_−), 0.75 (s, 3H, 18-CH_3_), 0.87 (m, 2H, −CHCH_2_CH_2_−), 1.11 (s, 3H, 19-CH_3_), 2.76 (s, 1H, −N(CH)), 5.47 (s, 1H, −CONH−), 5.82 (s, 1H, 4-H), 7.41 (s, 1H, 5-pyrazole) ppm. 13C NMR (75 MHz, CDCl_3_) δ: 13.02, 13.46, 14.21, 17.94, 21.11, 24.38, 25.35, 31.14, 31.79, 33.6, 36.04, 38.63, 39.77, 41.42, 44.59, 50.6, 53.65, 55.55, 76.15, 76.57, 77, 111.71, 111.84, 127.16, 148.76, 171.95 ppm. HRMS (ESI): *m/z* [M + H]^+^. Calcd for C_24_H_33_N_3_O: 380.2628; Found: 380.2951.

#### 2.1.19. General procedure for the preparation of 17β-(Piperidine-1-carbonyl)- androst[3,2-c]pyrazole -4-ene (9 b)

Yield: 45.20% of pure product, m.p.:154–162 °C;^1^H NMR(CDCl_3_, 300 MHz) δ: 0.77 (s, 3H, 18-CH_3_), 0.95 (s, 3H, 19-CH_3_), 3.56 (m, 4H,-N(CH_2_)_2_), 6.18 (s, 1H, 4-H), 6.54 (s, 1H, –CONH–), 7.22 (s, 1H, 5-pyrazole) ppm. 13C NMR (75 MHz, CDCl_3_) δ: 13.01, 13.42, 14.2, 17.95, 22.12, 24.39, 25.36, 31.15, 31.79, 33.58, 36.06, 38.62, 38.65, 39.73, 41.37, 44.55, 50.64, 53.67, 55.60, 92.74, 111.72, 112.07, 166.15 ppm. HRMS (ESI): *m/z* [M + H]^+^. Calcd for C_26_H_37_N_3_O_2_: 408.2940; Found: 408.3009.

#### 2.1.20. General procedure for the preparation of 17β-(N,N-diethy-carboxamide)-androst[3,2-c]pyrazole-4-ene (9c)

Yield: 47.91% of pure product, m.p.:111–118 °C; ^1^H NMR(CDCl_3_, 300 MHz) δ: 0.77 (s, 3H, 18-CH_3_), 3.01 (m, 4H, –NCH_2_), 3.76 (m, 4H, –NCH_2_), 6.18 (s, 1H, 4-H), 0.95 (s, 3H, 19-CH_3_), 7.21 (s, 1H, 5-pyrazole) ppm. 13C NMR (101 MHz, CDCl_3_) δ: 13.07, 18.03, 21.25, 23.57, 24.44, 31.33, 32.38, 35.44, 35.73, 37.57, 38.2, 39.88, 40.56, 43.57, 53.1, 55.5, 55.87, 55.97, 57.03, 76.71, 77.03, 77.34, 106.37, 111.41, 111.94, 120.66, 123, 131.44, 147.75, 149.13, 165.39, 169.76, 172.47, 188.95 ppm. HRMS (ESI): *m/z* [M + H]^+^. Calcd for C_29_H_37_N_3_O: 396.2941; Found: 396.3013.

#### 2.1.21. General procedure for the preparation of 17β-(N-(3,4-dimethoxyphenethyl)-carboxamide)-androst[3,2-c]pyrazole-4-ene(9d)

yield: 37.40%of pure product, m.p.:137–145 °C; ^1^H NMR(CDCl_3,_ 300 MHz) δ: 0.65 (s, 3H, 18-CH_3_), 0.87 (s, 3H, 19-CH_3_), 3.74 (s, 6H, −OCH_3_), 6.08 (s, 1H, 4-H), 6.79(m, 2H, Ar-H), 7.32(s, 1H, -CONH), 7.45 (s, 1H, 5-pyrazole), 12.15(s, 1H, 1-pyrazole)ppm. 13C NMR (CDCl_3,_ 75 MHz) δ: 5.85, 6.46, 12.66, 17.55, 22.04, 22.49, 23.91, 30.82, 31.89, 35.9, 37.04, 37.87, 39.39, 43.2, 52.58, 55.2, 55.7, 56.2, 105.9, 122.5, 164.75, 169.43, 173.44, 188.59 ppm. HRMS (ESI): *m/z* [M + H]^+.^Calcd for C_31_H_41_N_3_O_3:_ 504.3156; Found: 504.3225.

#### 2.1.22. General procedure for the preparation of 17β-(N-cyclopropylamino-carbonyl)-androst[3,2-c]pyrazole-4-ene (9e)

Yield: 73% of pure product, m.p.:91–97 °C; ^1^H NMR(CDCl_3_, 300 MHz) δ: 0.66 (s, 3H, 18-CH_3_), 0.86 (s, 3H, 19-CH_3_), 1.67 (m, 6H, −CH(CH_3_)_2_), 6.08 (s, 1H, 4-H), 7.31(s, 1H, −CONH),7.50 (s, 1H, 5-pyrazole), 12.22 (s, 1H, 1-pyrazole) ppm. 13C NMR (CDCl_3_, 75 MHz) δ: 5.85, 6.31, 6.47, 12.68, 18.05, 20.63, 22.43, 23.04, 23.9, 32.12, 32.14, 35.08, 37.84, 40.26, 41.37, 43.19, 44.53, 44.59, 44.9, 44.9, 45.07, 52.55, 52.55, 52.58, 53.1, 55.06, 56.2, 119.83, 124.2, 140.74, 171.28, 173.43 ppm. HRMS (ESI): *m/z* [M + H]^+^. Calcd for C_29_H_37_N_3_O: 396.2941; Found: 396.3013.

### 2.2. Biological activity

#### 2.2.1. Cell lines and culture conditions

Human prostate carcinoma 22RV1 (CRL-2505, ATCC) and PC-3 (CRL-1435, ATCC) cells were cultured in RPMI-1640 medium (Invitrogen), supplemented with 10% (v/v) foetal calf serum (FCS) (Invitrogen), 1% (v/v), penicillin and streptomycin (Invitrogen) at 37 °C with 5% CO_2_ in a humidified atmosphere. Fresh medium was given every second day and on the day before the experiments were done. Cells were passaged at preconfluent densities, using a solution containing 0.05% trypsin.

#### 2.2.2. Cell viability assay (MTT)

The anti-cancer activity *in vitro* was measured using the MTT assay. Exponentially growing cells were harvested and plated in 96-well plates at a concentration of 1 × 10^4^ cells/well. After 24 h incubation at 37 °C under a humidified 5% CO_2_ to allow cell attachment, the cells in the wells were respectively treated with target compounds at various concentrations for 48 h. The concentration of DMSO was always kept below 0.5%, which was found to be nontoxic to the cells. A solution of 3–(4,5-dimethylthizao1-2-y1)-2,5-diphenyltetrazolium bromide (MTT), was prepared at 5 mg/ml in phosphate buffered saline (PBS: 1.5 mM KH_2_PO_4_, 6.5 mM Na_2_HPO_4_, 137 mM NaCl, 2.7 mM KCl; pH 7.4). Of this solution 20 µl was added to each well. After incubation for 4 h at 37 °C in a humidified incubator with 5% CO_2_, the medium/MTT mixtures were removed, and the formazan crystals formed by the mitochondrial dehydrogenase activity of vital cells were dissolved in 100 µl of DMSO per well. The absorbance of the wells was read with a microplate reader at 570 nm. Effects of the drug cell viability were calculated using cell treated with DMSO as control.

#### 2.2.3. Enzyme inhibition test

Female rats were sacrificed and liver were taken within 5 min. All the following steps were performed at 0–4 °C. 1 g liver was cut into 3 mm pieces and added to 3 times volume of PBS. The tissue was homogenized and centrifuged at 10,000 g for 30 min. The supernatant was collected and centrifuged repeatedly at 100,000 for 1 h. The pellet obtained was resuspended in phosphate buffer (contained 30% glycerol, 1:3 v/v) and stored at −70 °C. The incubation mixture contained 50 µL enzyme solution, 6 µL NADPH (80 µM), 6 µL T (20 µM), and 108 µL buffer with or without test compound. The negative control contained 50 µL enzyme solution, 6 µL NADPH (80 µM) and 114 µL buffer. The incubation was carried out for 30 min at 37 °C. The fluorescence decay rate was tested in 10 min at 37 °C.

### 2.3. Molecular modelling

The molecular modelling was performed with Discovery Studio.3.0/CDOCK protocol (Accelrys Software Inc.). The crystal structures of AR (PDB code: 2PNU) were downloaded from Protein Data Bank. Compound **9d** was drowned and optimize using Hyperchem v7.0. The protein and ligands were optimized and charged with CHARMm force field to perform docking. Up to 10 conformations were retained and binding modes presented graphically are representative of the highest-scored conformations.

## 3. Results and discussion

### 3.1. Chemistry

**Scheme 1.**
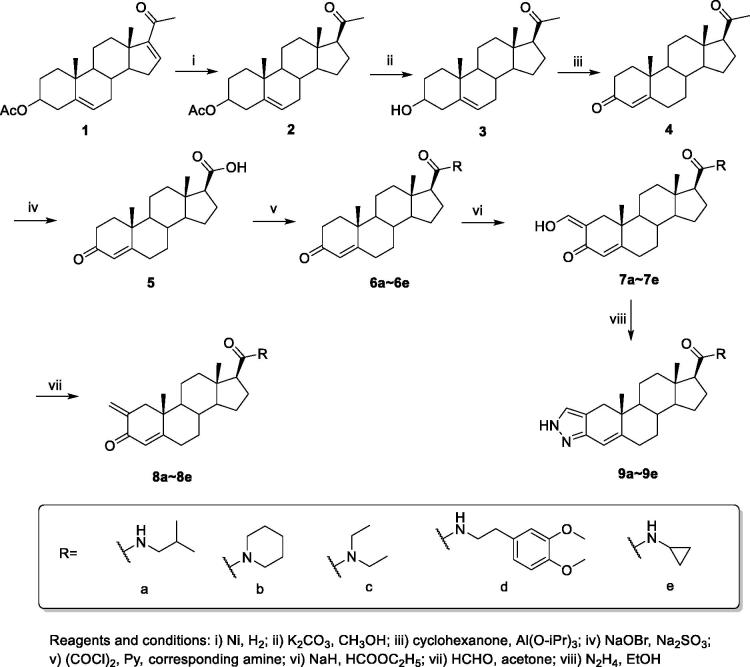
The synthesis of compound **7a–9e**.

Taking commercial available compound 16-dehydropregnenolone acetate (**1**) as starting material, the targeting compounds were synthesized through 8 steps (Scheme 1). Compound **2** was afforded by the selective reduction of **1** with Raney Ni in EtOH. After filtration, the hydrolysis of the acetate group at C-3 was produced by directly adding KOH to resulting filtrate to obtain compound **3.** The resulting secondary alcohol at C-3 was oxidized to the α,β-unsaturated ketone compound **4** by Oppenauer oxidation using aluminium isopropoxide and cyclohexanone in refluxing toluene. The17-amide derivatives **6a–6e** were then obtained by treating **5** with bromine and sodium hydroxide followed by acylation with different amide, respectively. The 2-hydroxymethylene derivatives **7a–7e** were prepared by the formulation of **6a–6e** with NaH and HCOOEt in toluene. Compound **8a–8e** were then obtained by the reduction of HCHO. The cyclisation of compound **8a–8e** under hydrazinium hydroxide in EtOH eventually afford compound **9a–9e**. The representative ^1^H NMR, ^13^C NMR and MS spectra are depicted in the supplementary material.

### 3.2. Anti-proliferative activity

To investigate their anti-cancer effects, all the synthesized compounds were tested for their anti-proliferative activities in 2 PCa cell lines by taking finasteride and flutaminde as positive control.

**Figure 3.**
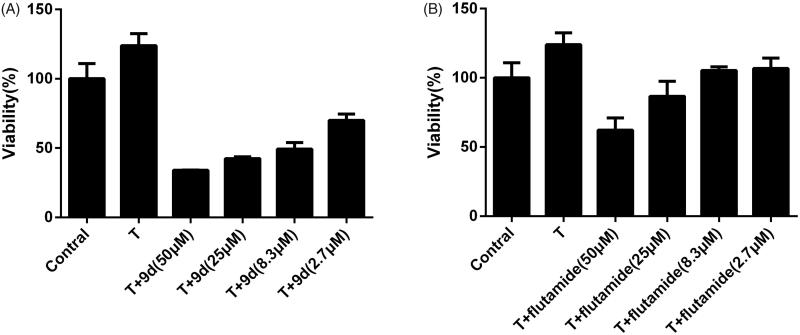
Effects of compounds **9d** (A) and flutamide (B) in cell viability of T-treated 22RV1 cells. 22RV1 cells were cultured with different concentrations of tested compounds and 0.1 μM T. Values are mean ± SD (*n* = 3). ***p < 0.001 versus T group.

The anti-proliferative activities were firstly evaluated against 22RV1 stimulated by 0.1 µM T or not. As shown in Table 1, most compounds presented better growth inhibition activity in T-stimulated group than the non-stimulated group, suggesting that the anti-proliferative effects were attributed to androgen-related signalling pathway due to their competitive binding to AR with T. For T-stimulated group, most compounds displayed good activity with the IC_50_ less than 30 µM. It was obvious that androst[3,2-c]pyrazoleis derivatives (**9a∼9e**) were more potent than others with their IC_50_ lower than 20 µM. Among them, compound **9d** was the most potent with IC_50_ of 8.85 µM, much better than the positive control. Although androst[3,2-c]pyrazoleis common in anabolic steroid, luckily these derivatives shown no androgen agonistic activity. The phenethylamino substituted compounds (**7d, 8d**, and **9d**) and piperidine substituted compounds (**7b, 8b**, and **9b**) were more efficient than others. The small sterically hindered derivatives (**7a, 8a**, and **9a**) showed lower growth inhibition activity, which proved that increasing of the steric hindrance of amide substitution led to better inhibitory activity. As presented in Figure 3, 0.1 µm of T was selectedas the most adequate to induce proliferation of 22RV1 cells. Results shown in Figure 3(A,B) indicate that compound **9d** and flutamide inhibited cell proliferation in a dose-dependent manner. Compound **9d** caused a much more significant decrease in cell viability for all concentrations than flutamide.

**Table 1.** The anti-proliferation activities of synthesized compounds against 22RV1.

To investigate the selectivity and safety of the synthesized compounds, the anti-proliferative activity was further assessed against PC-3 cell line. Since PC-3 is AR-independent cell, the anti-proliferative effect was mainly attributed to the non-androgen signal pathway mechanism or cytotoxicity. As shown in Table 2, the majority of compounds presented much weaker anti-proliferation activity toward PC-3 with IC_50_ twice higher than the IC_50_ toward 22RV1, which proves their good androgen antagonist activity and safety. The highest IC_50_ was observed among androst[3,2-c]pyrazoleis (**9a∼9e**), in which compound **9d** was the most efficient with IC_50_ 9.74 times higher than its IC_50_ toward 22RV1. The low toxicity and good selectivity warrant its further development for identifying more potent anticancer agents.

**Table 2.** The anti-proliferation activities of synthesized compounds against PC-3.

### 3.3. Enzyme inhibitory activity

The inhibitory activities of the synthesized compounds were then assessed towards the 5α-reductase 1 and 2 isozymes, respectively. Finasteride was used as positive control. As shown in Table 3, many compounds exhibited good inhibitory activities with IC_50_ lower than 1 µM, comparable to the positive control finasteride. For most compounds, the inhibitory activities towards the type 1 isozyme were slightly higher than type 2. Among them, the androst[3,2-c]pyrazole derivatives (**9a–9e**) were much more potent with the inhibition rate higher than 50%. The 2-methylene derivatives (**8a–8e**) presented a much inferior inhibitory activity to the others, which suggested that the electron-withdrawing group at 2-C was essential for the inhibitory activity. Consistent with the anti-proliferative result, it was also found that the increasing of the steric hindrance of amide substitution led to better inhibitory activity. The compounds with phenethylamino substitution (**7d, 8d, and 9d**) showed better inhibitory activities than others, in which, the most potential compounds **9d,** with IC_50_ of 0.09 and 0.08 µM for 1 and 2 isozymes, respectively, was better than the positive control finasteride.

**Table 3.** The inhibitory activities of synthesized compounds towards two 5α-reductaseiozymes.

Compound	Type 1 (pH 6.6)		Type 2 (pH 5.5)	
	Inh% (1 μM)	IC_50_ (μM)	Inh% (1μM)	IC_50_ (μM)
**7a**	24.96 ± 9.68	–	24.16 ± 1.13	–
**7b**	52.20 ± 12.33	–	49.91 ± 3.08	–
**7c**	27.92 ± 4.01	–	24.52 ± 8.47	–
**7d**	66.42 ± 4.37	0.58 ± 0.06	69.03 ± 9.30	0.41 ± 0.03
**7e**	54.70 ± 5.94	–	45.75 ± 9.86	–
**8a**	31.14 ± 6.49	–	19.88 ± 12.91	–
**8b**	35.76 ± 9.58	–	21.17 ± 1.31	–
**8c**	25.82 ± 4.22	–	18.29 ± 4.61	–
**8d**	24.82 ± 9.02	–	26.06 ± 10.34	–
**8e**	34.09 ± 10.05	–	20.94 ± 10.06	–
**9a**	53.15 ± 9.71	–	48.69 ± 3.08	–
**9b**	59.25 ± 2.28	0.63 ± 0.14	49.62 ± 5.75	1.01 ± 0.06
**9c**	68.02 ± 11.96	0.52 ± 0.06	63.56 ± 12.47	0.41 ± 0.14
**9d**	80.14 ± 6.73	0.09 ± 0.02	71.92 ± 3.40	0.08 ± 0.07
**9e**	70.77 ± 9.74	0.23 ± 0.09	79.41 ± 2.47	0.21 ± 0.09
Finasteride	73.69 ± 4.22	1.04 ± 0.08	70.04 ± 4.12	0.04 ± 0.02

### 3.4. Molecular docking studies

**Figure 4.**
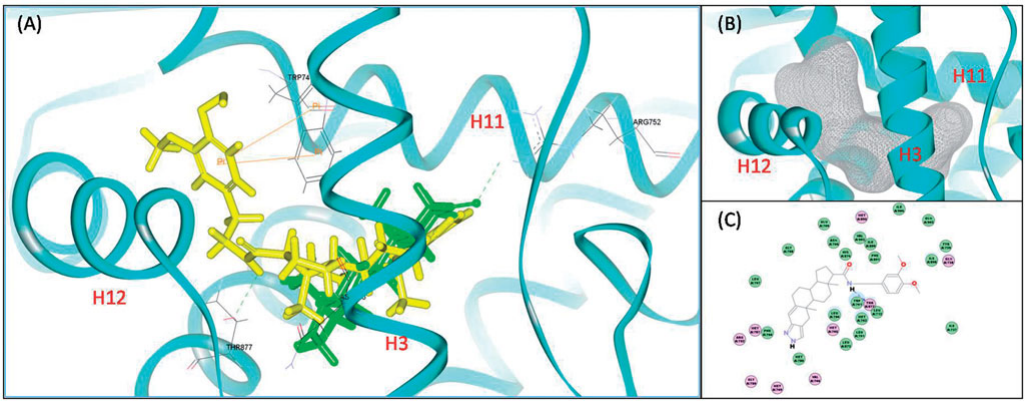
(A) The detailed docking result of compound **9d** (yellow) and DHT (green) with AR. The hydrogen bonds were indicated by green-dotted lines. The Pi–Pi interactions were indicated by orange lines. (B) The expended binding cavity (grey) of AR. (C) The 2D diagram of **9d**-AR binding mode.

To further rationalize the prospective activities of designed compound against AR, molecular docking studies were performed using the Discovery Studio 3.0/CDOCKER protocol. The docking orientation and interactions of **9d** and DHT within the ligand binding domain (LBD) of AR (2PNU, PDB) are shown in Figure 4. As shown in Figure 4(B), AR was forced to expands its binding cavity and maintain an antagonistic conformation because of the steric hindrance that impede the transposition of H12. As an antagonist conformation of AR, there is only one hydrogen bond between DHT and AR forming with Arg752, but lacking the “latch” effect of the two hydrogen bond with Thr877 and Asn705 (Figure 4(A)). For **9d**, its steroidal-core was favorably positioned similar to DHT while the bulky substitution at 17-amide pointing to H12 (Figure 4(A)). The detailed docking analysis in 2D diagram (Figure 4(C)) showed that the aromatic group at the substitution of C-17 established a Pi-Pi interaction with Trp741 when the amide group picked up hydrogen bonds with Thr877.

## 4. Conclusion

In this study, a series of substituted androst-17β-amide derivatives were designed and synthesized. Biological evaluation was performed on their anti-proliferative activities against 2 PCa cell lines. The result anti-proliferative effects against 22RV1 showed that most compounds exhibited better anti-proliferative activity in T-stimulate group, which was attributed to androgen-related signalling pathway due to their competitive binding to AR. Among them, compound **9d** showed the best anti-proliferative activity with the IC_50_ of 8.85 µM, more potent than the positive control. Moreover, the investigation of anti-proliferative activity against PC-3 showed that the majority of compounds exhibited good selectivity and low toxicity against androgen-independent cells, suggesting their acceptable safety profiles as anti-tumour agents. In the further study of the inhibitory activity of the 5α-reductase 1 and 2 isozymes, androst[3,2-c]pyrazole derivatives **(9a–9d)** were observed as more potent inhibitors, which was consisted with their anti-proliferative activities. Among all the compounds, **9d** was found to be the most potential inhibitor with the IC_50_ of 0.09 and 0.08 µM, respectively. Overall, based on biological activities data, compound **9d** can be identified as potential dual 5α-reductase inhibitors and AR antagonists lead molecule which might be of therapeutic importance for PCa treatment.

## Supplementary Material

Supplemental Material
