# Supplementary material for: Design, synthesis, and biological evaluation of novel androst-17β-amide structurally related compounds as dual 5α-reductase inhibitors and androgen receptor antagonists
Source: J Enzyme Inhib Med Chem. 2019 Aug 30;34(1):1597–606. doi: 10.1080/14756366.2019.1654469 (PMC6735293; doi:10.1080/14756366.2019.1654469)

Supporting Information

Design, Synthesis and Biological Evaluation of Novel androst-17β-amide structurally related compounds as Dual 5α-reductase Inhibitors and Androgen Receptor Antagonists

Kejing Lao[a,b,c], Guoliang Xun[d], Xingchun Gou[a*], Hua Xiang[b,c*]

[a]*Institute of Basic and Translational Medicine & Shaanxi Key Laboratory of Brain Disorders, Xi'an Medical University, No.1 Xinwang Road, Xi'an, 710021, PR China*

[b]*Jiangsu Key Laboratory of Drug Design and Optimization , China Pharmaceutical University, 24 Tongjiaxiang, Nanjing 210009, PR China*

[c]*Department of Medicinal Chemistry, School of Pharmacy, China Pharmaceutical University, 24 Tongjiaxiang, Nanjing 210009, PR China*

[d]*Shanghai Abbisko Therapeutics Co., Ltd*

* Corresponding author. Tel.: +86 29 86177603; Fax:+86 29 86177603 (X. Gou) and Tel.: +86 025 83271096; Fax: +86 025 83271096 (H. Xiang). E-mail addresses: gxchun@126.com (X. Gou) and xianghua@cpu.edu.cn (H. Xiang).

7a

2A
L0538017 CDCL3 1HNMR AV300

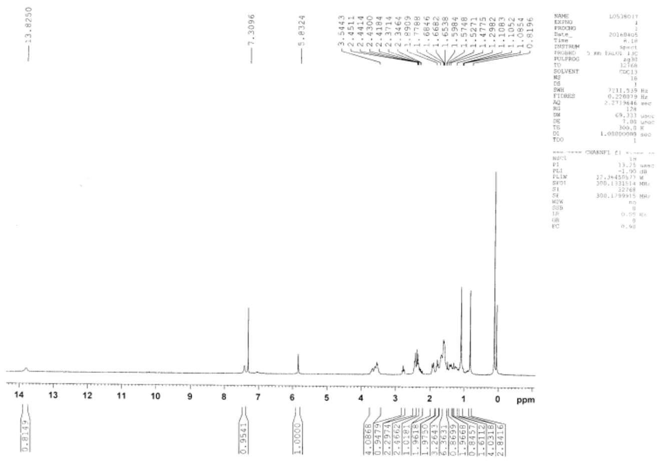

L0538017 C13-NMR CDCL3 303K AV-300

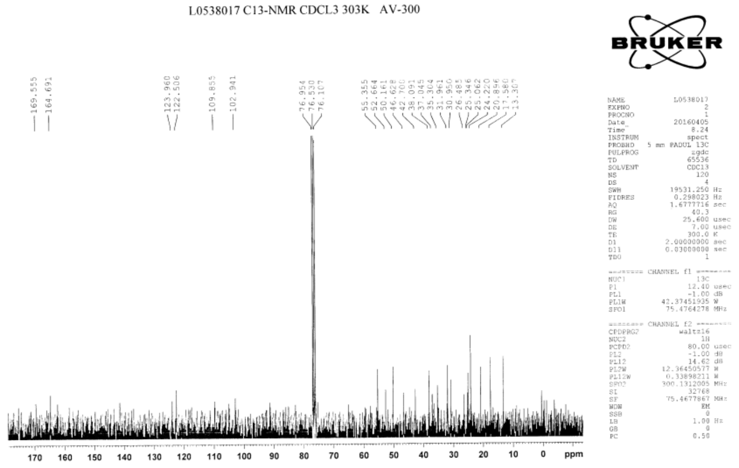

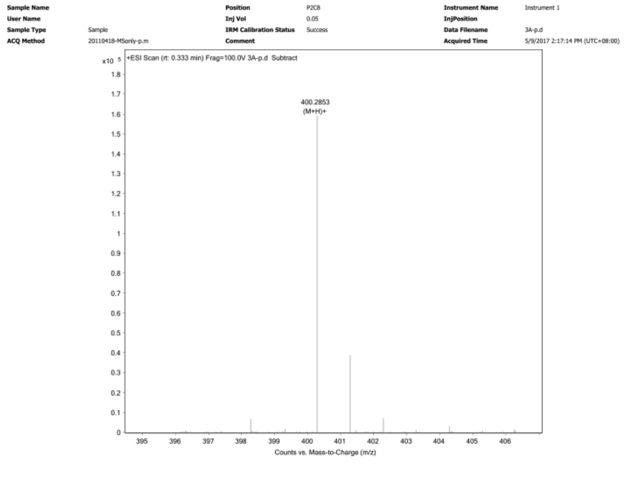

7b

2B
L0538025 CDCL3 1HNMR AV300

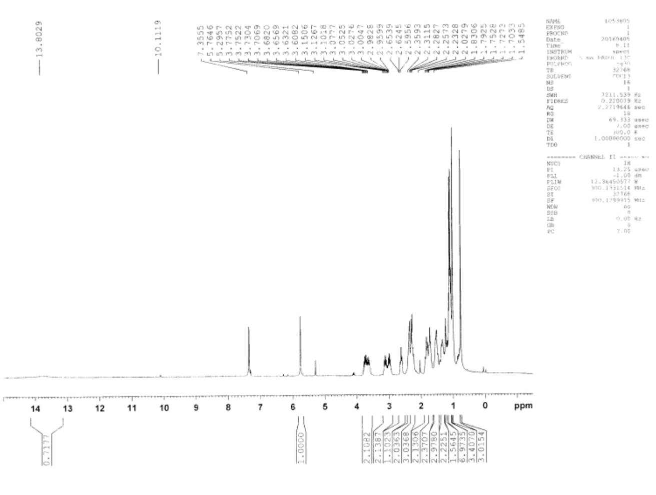

L0538025 C13-NMR CDCL3 303K AV-300

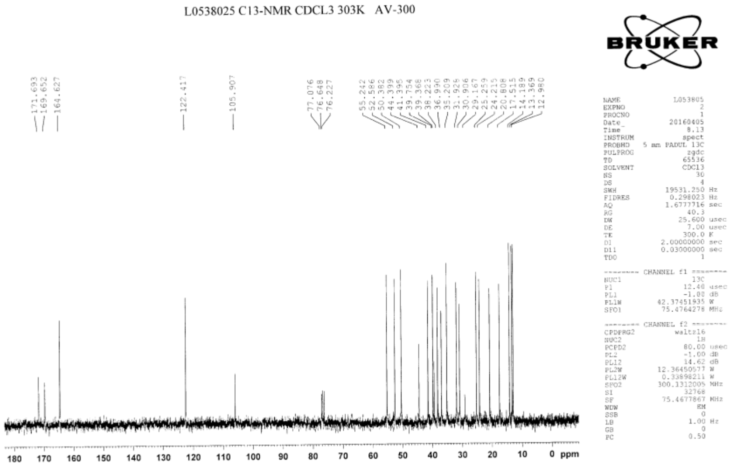

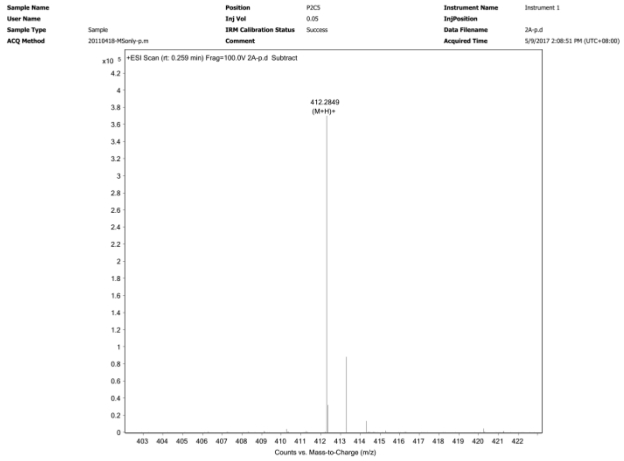

7c

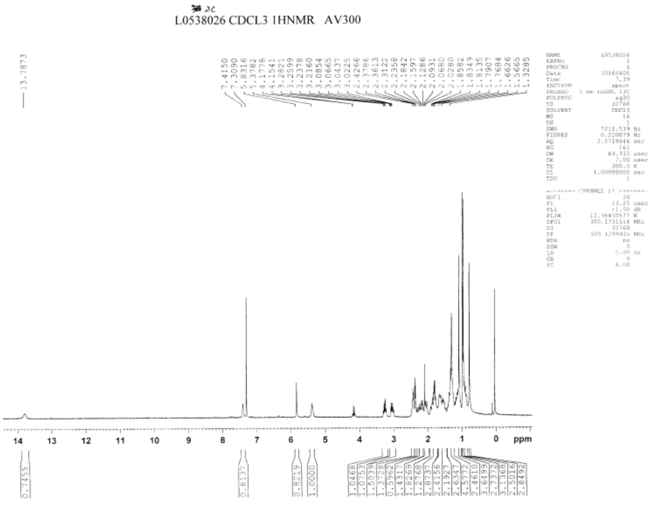
L0538026 CDCL3 1HNMR AV300
ppm

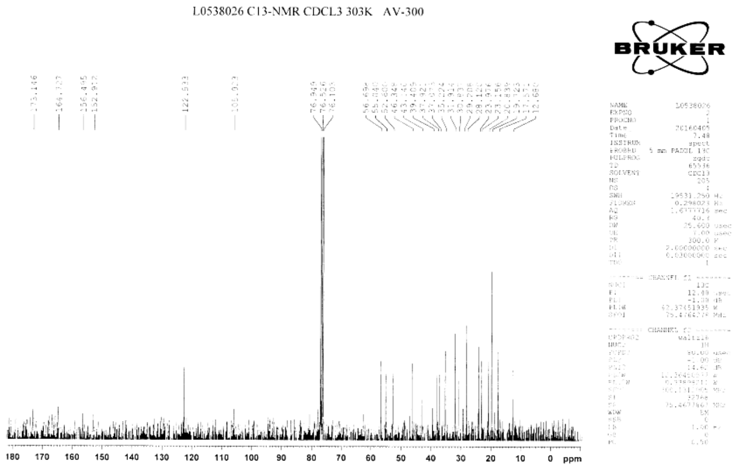
L0538026 C13-NMR CDCL3 303K AV-300
BRUKER
ppm

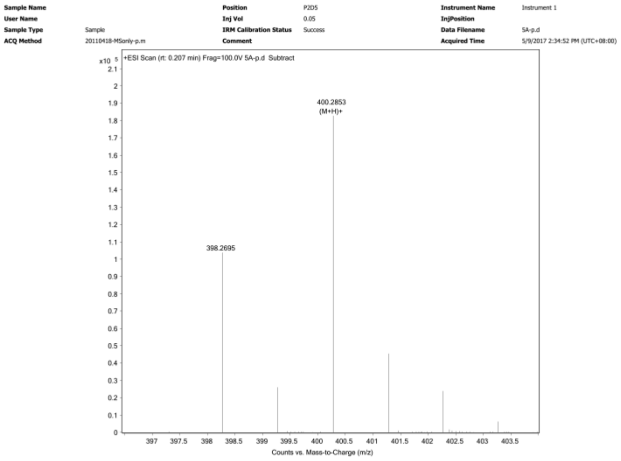
Sample Name
User Name
Sample Type Sample
ACQ Method 20110418-MSonly-p.m
Position P2D5
Inj Vol 0.05
IRM Calibration Status Success
Comment
Instrument Name Instrument 1
InjPosition
Data Filename 5A-p.d
Acquired Time 5/9/2017 2:34:52 PM (UTC+08:00)
+ESI Scan (rt: 0.207 min) Frag=100.0V 5A-p.d Subtract
400.2853
(M+H)+
398.2695
Counts vs. Mass-to-Charge (m/z)

7d

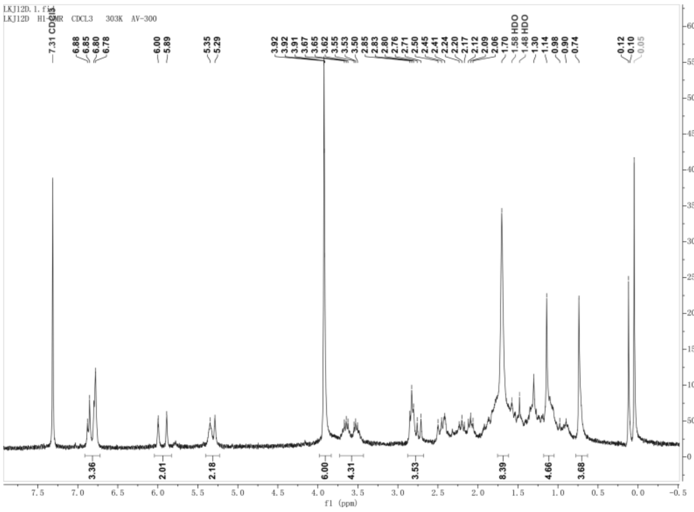
LKJ12D.1.fid
LKJ12D H1-NMR CDCL3 303K AV-300
f1 (ppm)

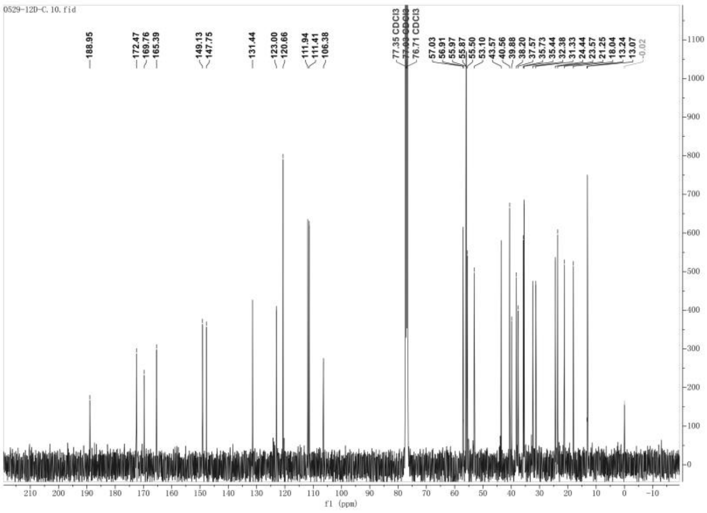
0529-12D-C.10.fid
f1 (ppm)

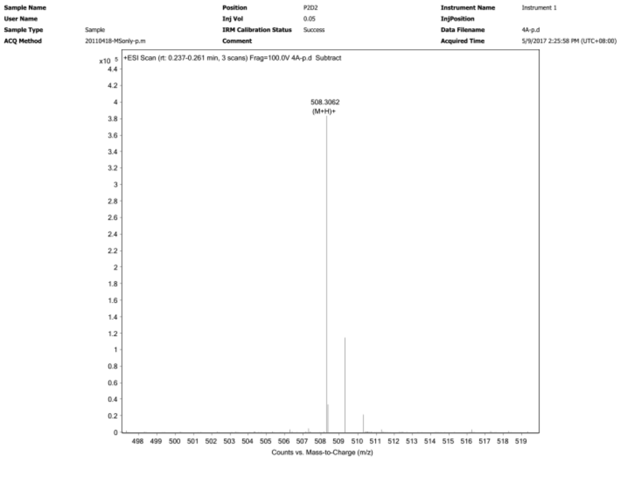
Sample Name
User Name
Sample Type Sample
ACQ Method 20110418-MSonly-p.m
Position P2D2
Inj Vol 0.05
IRM Calibration Status Success
Comment
Instrument Name Instrument 1
InjPosition
Data Filename 4A-p.d
Acquired Time 5/9/2017 2:25:58 PM (UTC+08:00)
+ESI Scan (rt: 0.237-0.261 min, 3 scans) Frag=100.0V 4A-p.d Subtract
508.3062
(M+H)+
Counts vs. Mass-to-Charge (m/z)

7e

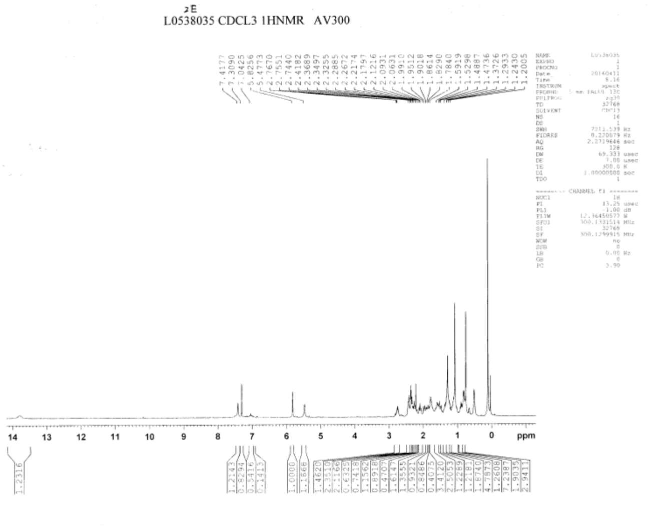
L0538035 CDCL3 1HNMR AV300
ppm

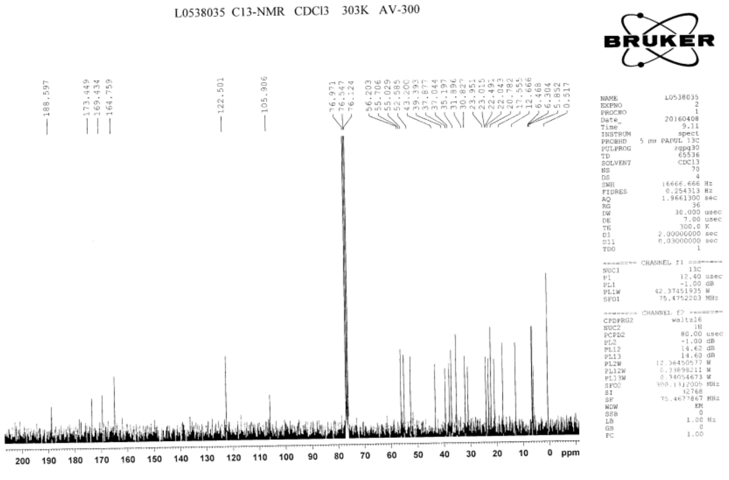
L0538035 C13-NMR CDCl3 303K AV-300
BRUKER
ppm

8a

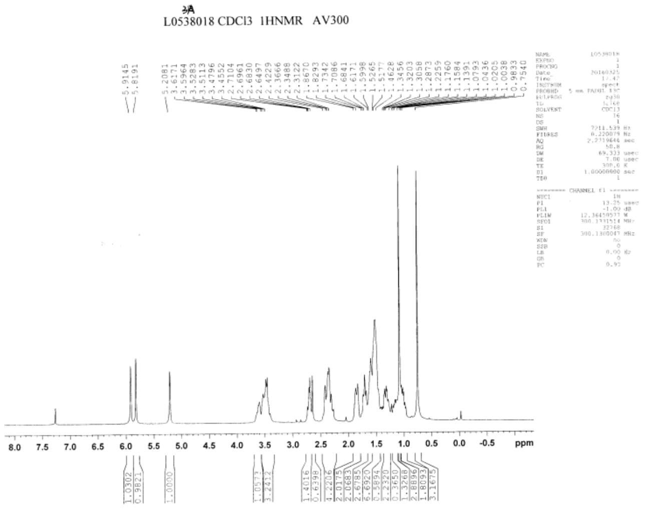
L0538018 CDCl3 1HNMR AV300
ppm

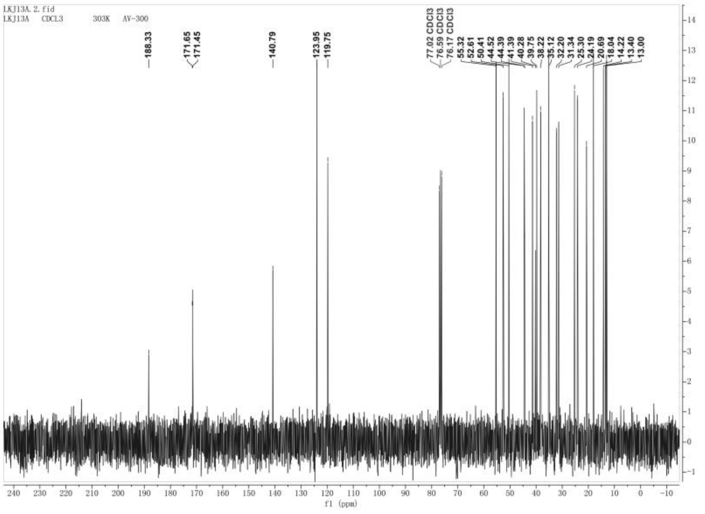
LKJ13A.2.fid
LKJ13A CDCL3 303K AV-300
f1 (ppm)

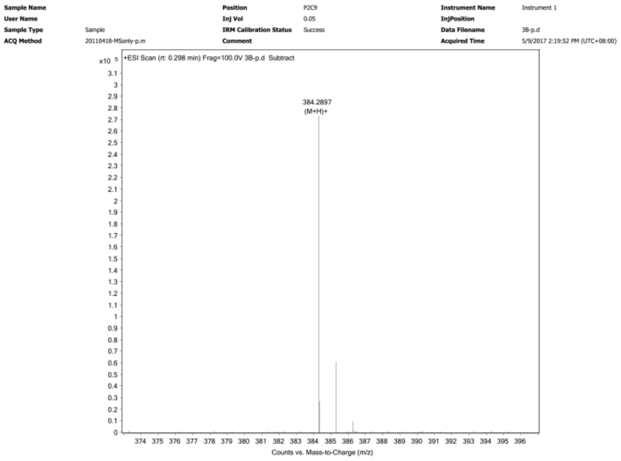
Sample Name
User Name
Sample Type Sample
ACQ Method 20110418-MSonly-p.m
Position P2C9
Inj Vol 0.05
IRM Calibration Status Success
Comment
Instrument Name Instrument 1
InjPosition
Data Filename 3B-p.d
Acquired Time 5/9/2017 2:19:52 PM (UTC+08:00)
+ESI Scan (rt: 0.298 min) Frag=100.0V 3B-p.d Subtract
384.2897
(M+H)+
Counts vs. Mass-to-Charge (m/z)

8b

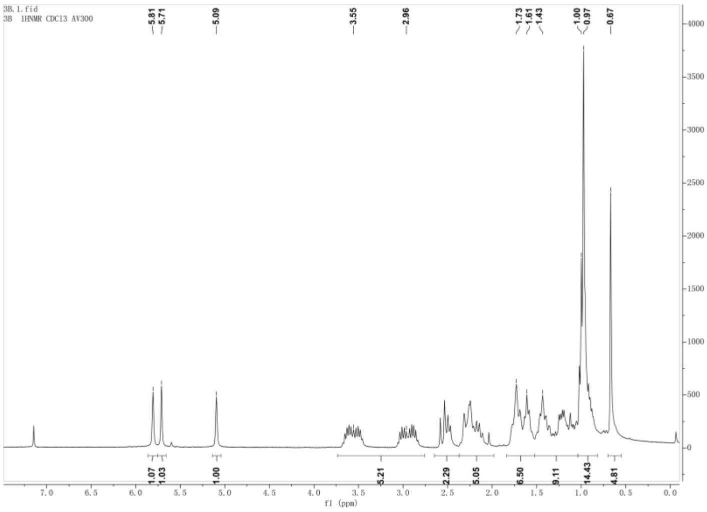
3B.1.fid
3B 1HNMR CDC13 AV300
f1 (ppm)

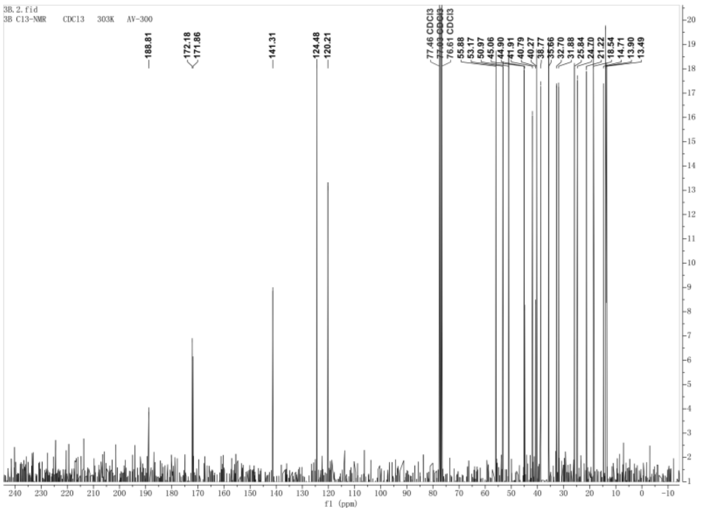
3B.2.fid
3B C13-NMR CDCl3 303K AV-300
188.81
172.18
171.86
141.31
124.48
120.21
77.46 CDCl3
76.61 CDCl3
f1 (ppm)

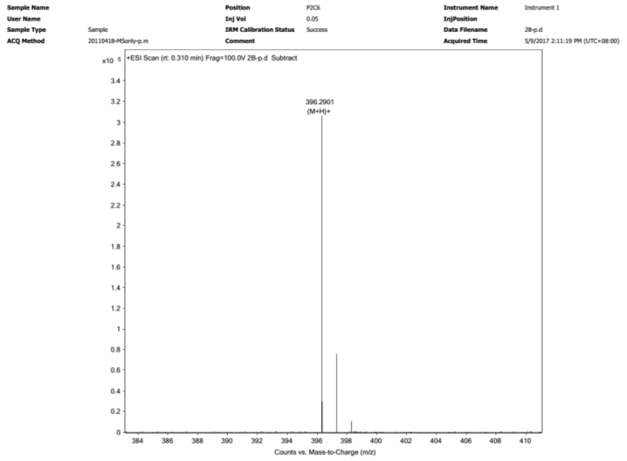
Sample Name
User Name
Sample Type Sample
ACQ Method 20110418-MSonly-p.m
Position P2C6
Inj Vol 0.05
IRM Calibration Status Success
Comment
Instrument Name Instrument 1
InjPosition
Data Filename 2B-p.d
Acquired Time 5/9/2017 2:11:19 PM (UTC+08:00)
+ESI Scan (rt: 0.310 min) Frag=100.0V 2B-p.d Subtract
396.2901
(M+H)+
Counts vs. Mass-to-Charge (m/z)

8c

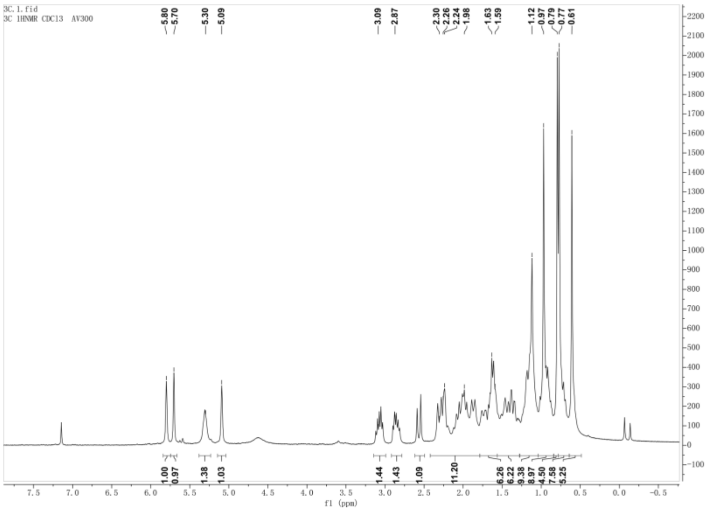
3C.1.fid
3C 1HNMR CDCl3 AV300
5.80
5.70
5.30
5.09
3.09
2.87
2.30
2.26
2.24
1.98
1.63
1.59
1.12
0.97
0.79
0.77
0.61
1.00
0.97
1.38
1.03
1.44
1.43
1.09
11.20
6.26
6.22
9.38
8.97
4.50
7.58
5.25
f1 (ppm)

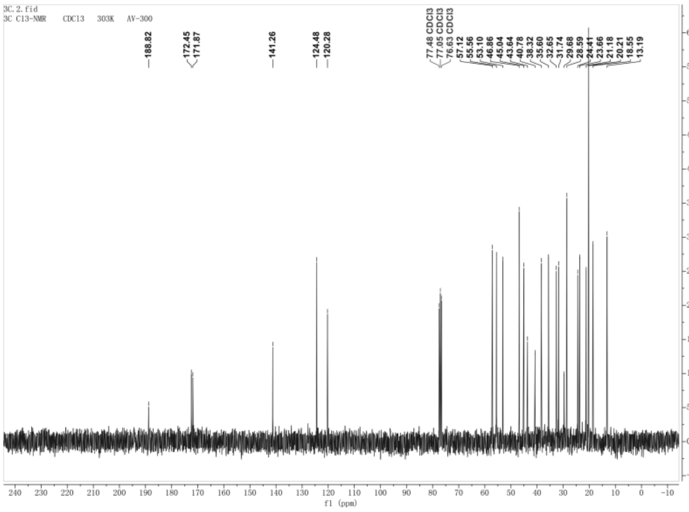

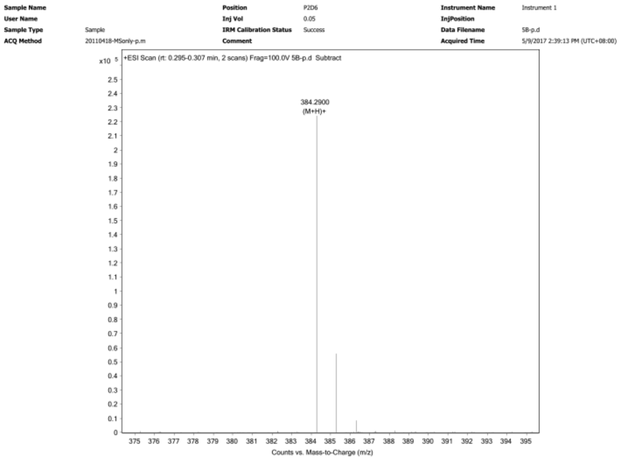

8d

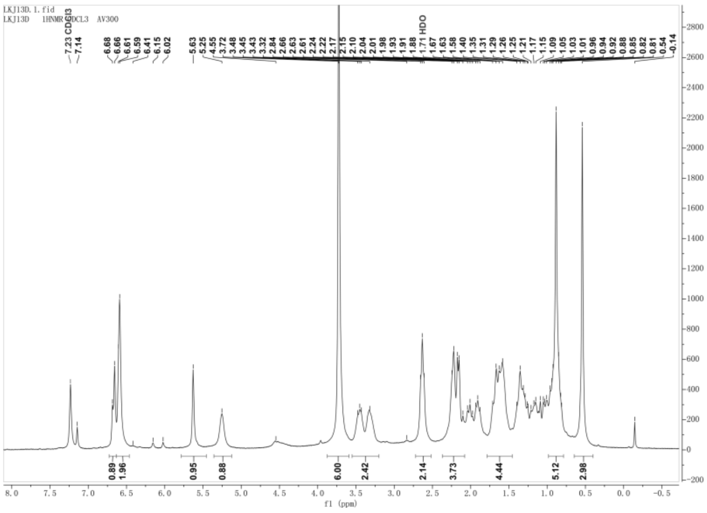

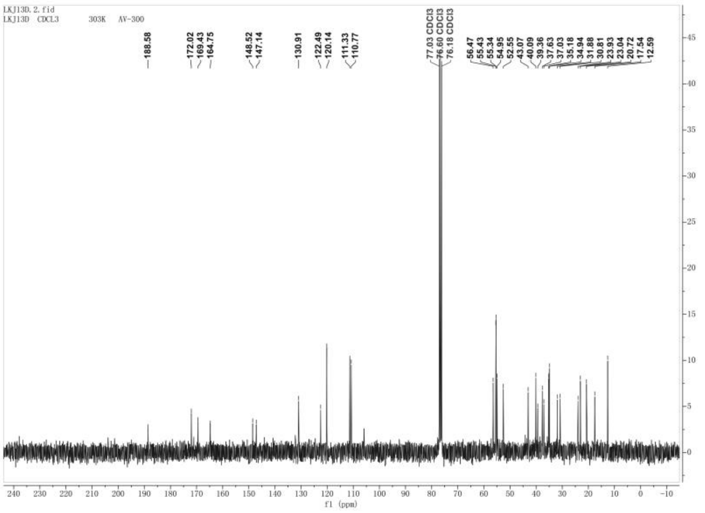
LKJ13D.2.fid
LKJ13D CDCL3 303K AV-300
188.58
172.02
169.43
164.75
148.52
147.14
130.91
122.49
120.14
111.33
110.77
77.03 CDCl3
76.60 CDCl3
76.18 CDCl3
56.47
55.43
55.34
54.95
52.55
43.07
40.09
39.36
37.63
37.03
35.18
34.94
31.88
30.81
23.93
23.04
20.72
17.54
12.59
f1 (ppm)

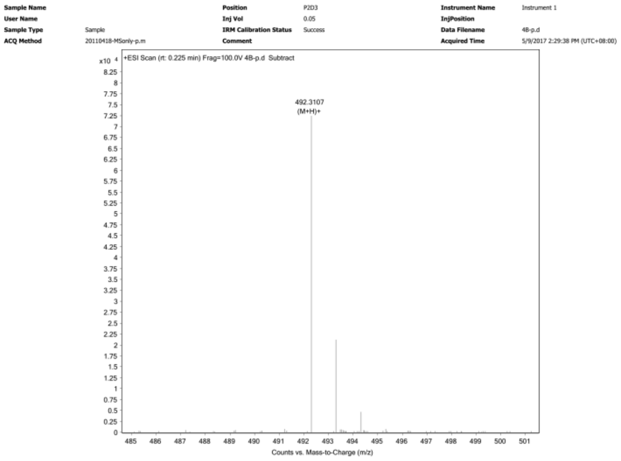
Sample Name
User Name
Sample Type Sample
ACQ Method 20110418-MSonly-p.m
Position P2D3
Inj Vol 0.05
IRM Calibration Status Success
Comment
Instrument Name Instrument 1
InjPosition
Data Filename 4B-p.d
Acquired Time 5/9/2017 2:29:38 PM (UTC+08:00)
+ESI Scan (rt: 0.225 min) Frag=100.0V 4B-p.d Subtract
492.3107
(M+H)+
Counts vs. Mass-to-Charge (m/z)

8e

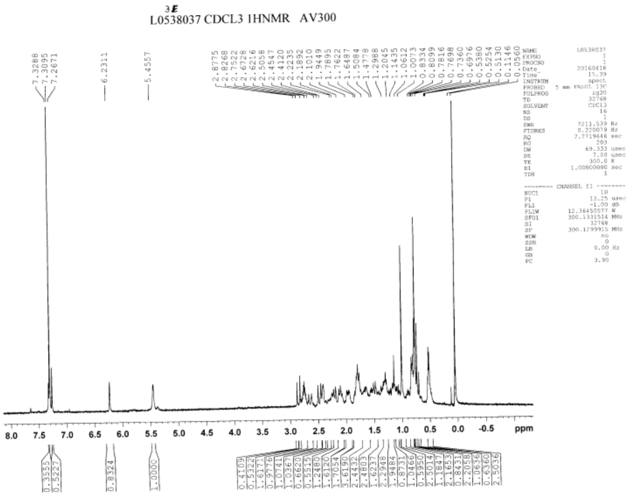
L0538037 CDCL3 1HNMR AV300
ppm

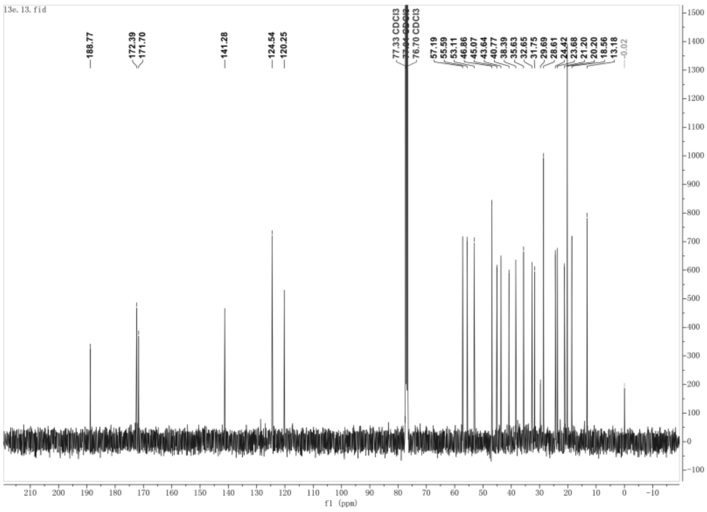
13c.13.fid
188.77
172.39
171.70
141.28
124.54
120.25
77.33 CDCl3
76.70 CDCl3
57.19
55.59
53.11
46.86
45.07
43.64
40.77
38.39
35.63
32.65
31.75
29.69
28.61
24.42
23.68
21.20
20.20
18.56
13.18
-0.02
f1 (ppm)

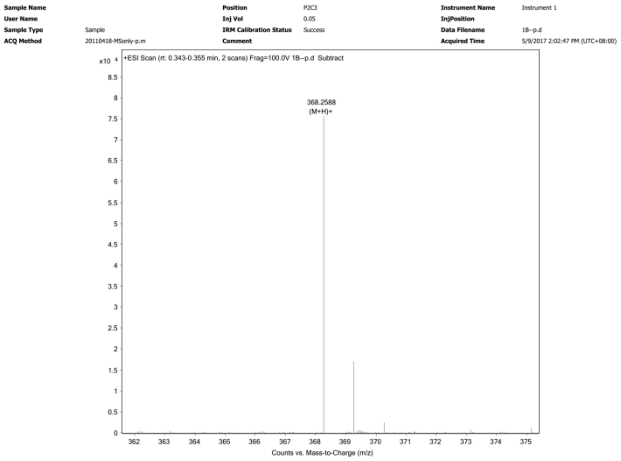
Sample Name
User Name
Sample Type Sample
ACQ Method 20110418-MSonly-p.m
Position P2C3
Inj Vol 0.05
IRM Calibration Status Success
Comment
Instrument Name Instrument 1
InjPosition
Data Filename 1B--p.d
Acquired Time 5/9/2017 2:02:47 PM (UTC+08:00)
+ESI Scan (rt: 0.343-0.355 min, 2 scans) Frag=100.0V 1B--p.d Subtract
368.2588
(M+H)+
Counts vs. Mass-to-Charge (m/z)

9a

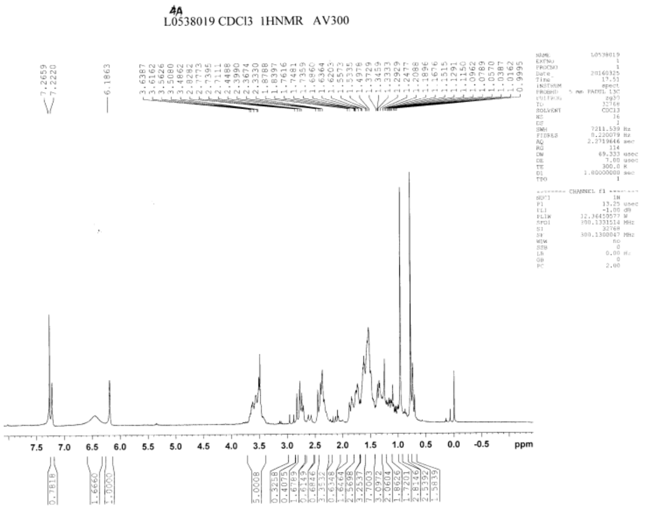
4A
L0538019 CDCl3 1HNMR AV300
ppm

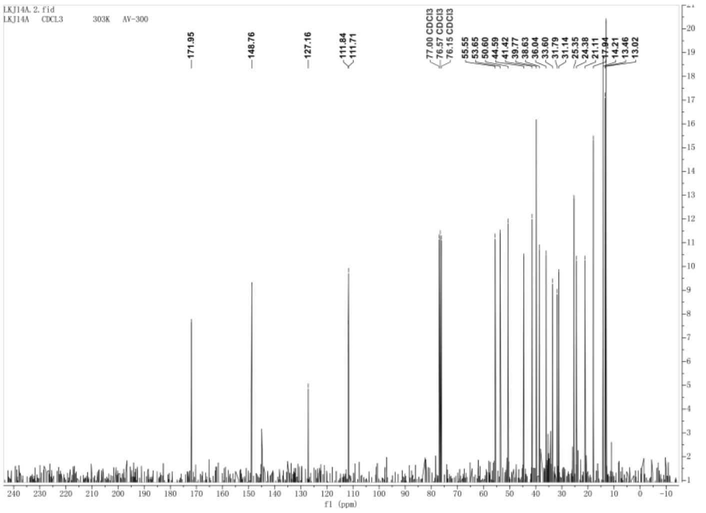
LKJ14A.2.fid
LKJ14A CDCL3 303K AV-300
171.95
148.76
127.16
111.84
111.71
77.00 CDCl3
76.57 CDCl3
76.15 CDCl3
55.55
53.65
50.60
44.59
41.42
39.77
38.63
36.04
33.60
31.79
31.14
25.35
24.38
21.11
17.94
14.21
13.46
13.02
f1 (ppm)

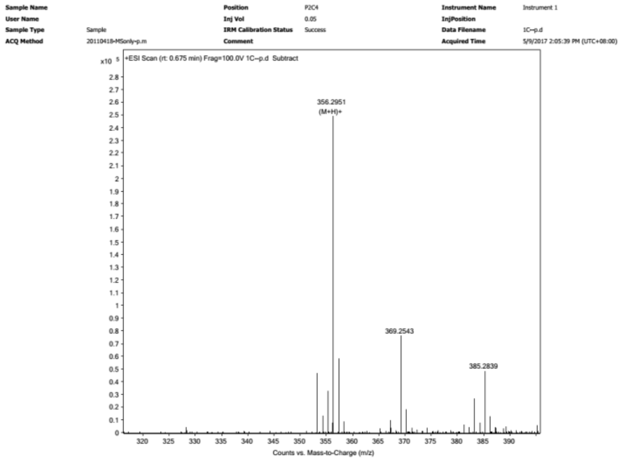
Sample Name
User Name
Sample Type Sample
ACQ Method 20110418-MSonly-p.m
Position P2C4
Inj Vol 0.05
IRM Calibration Status Success
Comment
Instrument Name Instrument 1
InjPosition
Data Filename 1C-p.d
Acquired Time 5/9/2017 2:05:39 PM (UTC+08:00)
+ESI Scan (rt: 0.675 min) Frag=100.0V 1C-p.d Subtract
356.2951
(M+H)+
369.2543
385.2839
Counts vs. Mass-to-Charge (m/z)

9b

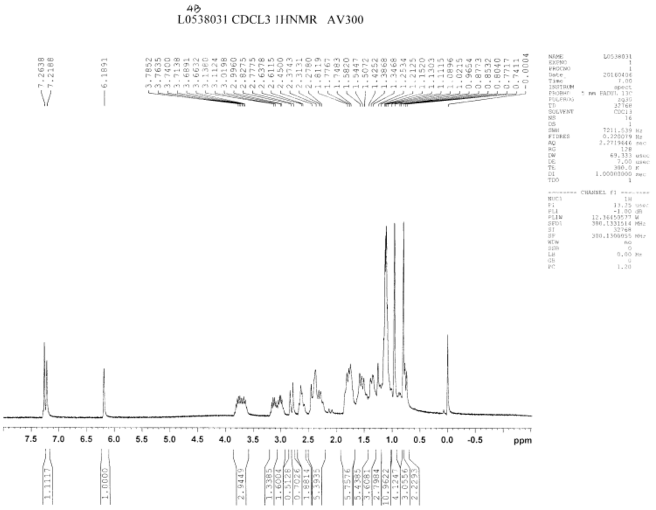
L0538031 CDCL3 1HNMR AV300
7.2638
7.2108
6.1891
ppm
1.1117
1.0000
2.9449

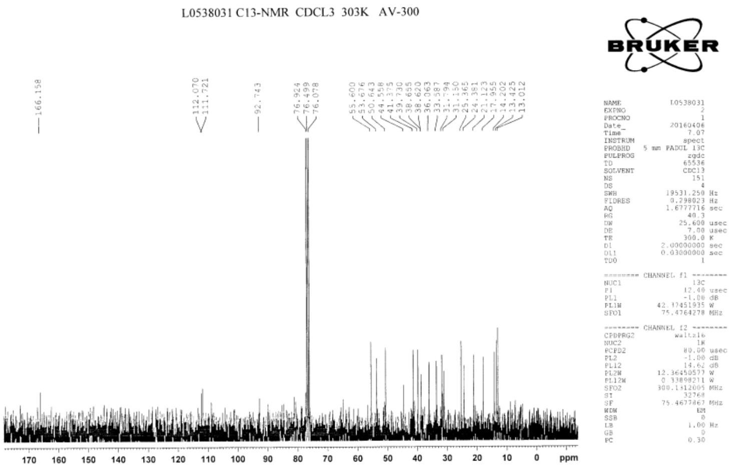

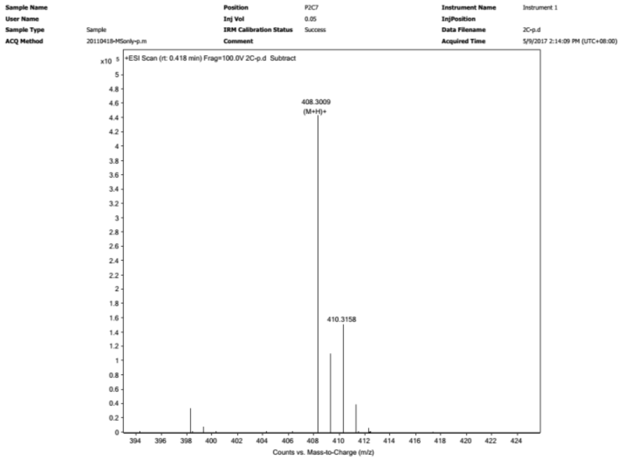

9c

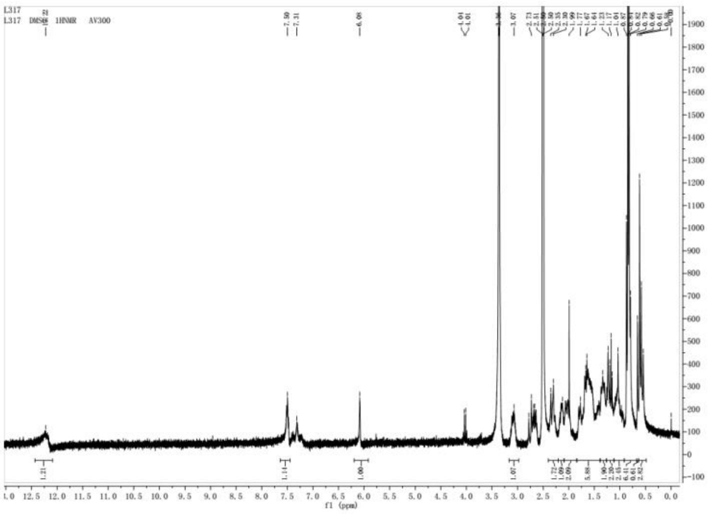

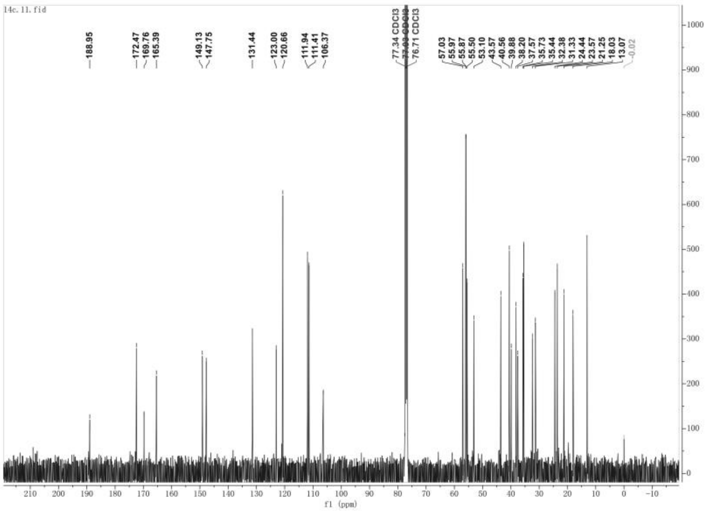

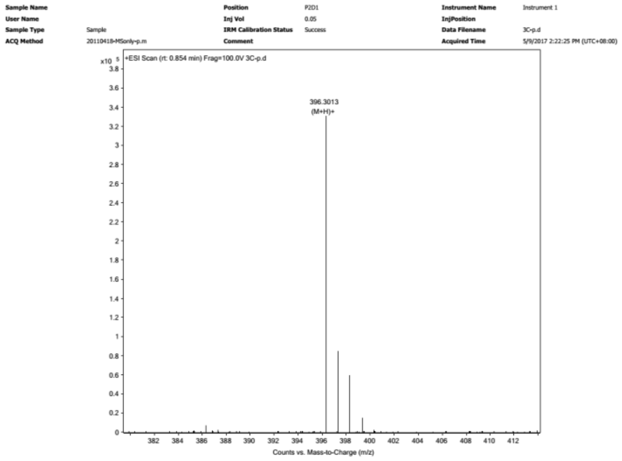

9d

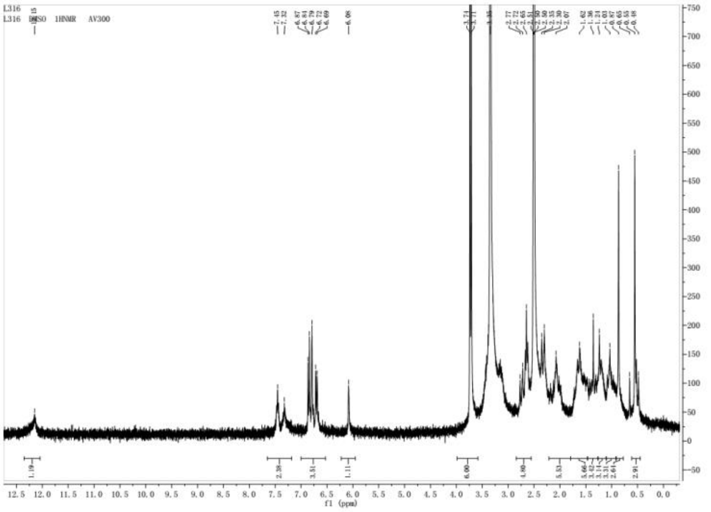

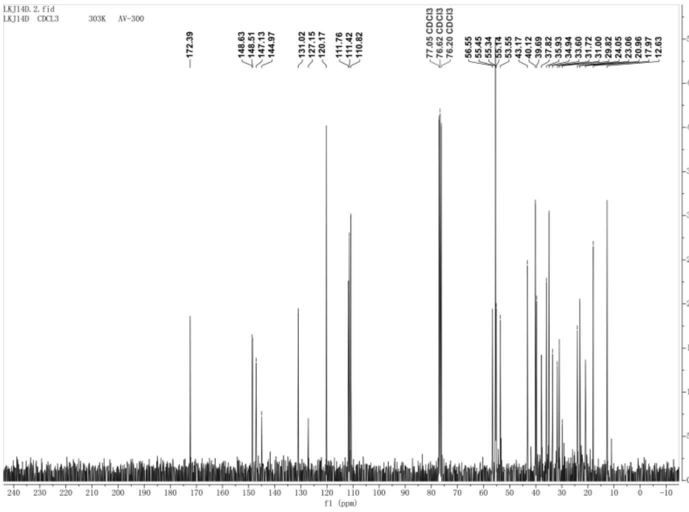
LKJ14D.2.fid
LKJ14D CDCL3 303K AV-300
172.39
148.63
148.51
147.13
144.97
131.02
127.15
120.17
111.76
111.42
110.82
77.05 CDCl3
76.62 CDCl3
76.20 CDCl3
56.55
55.45
55.34
55.14
53.55
43.17
40.12
39.69
37.82
35.93
34.94
33.60
31.72
31.00
29.82
24.05
23.06
20.96
17.97
12.63
f1 (ppm)

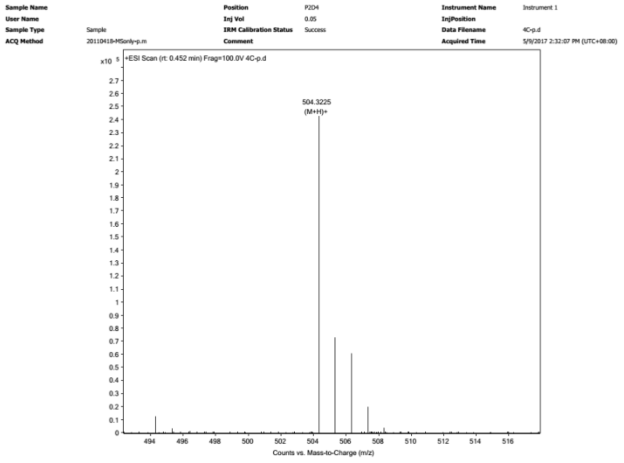
Sample Name
User Name
Sample Type Sample
ACQ Method 20110418-MSonly-p.m
Position P2D4
Inj Vol 0.05
IRM Calibration Status Success
Comment
Instrument Name Instrument 1
InjPosition
Data Filename 4C-p.d
Acquired Time 5/9/2017 2:32:07 PM (UTC+08:00)
+ESI Scan (rt: 0.452 min) Frag=100.0V 4C-p.d
504.3225
(M+H)+
Counts vs. Mass-to-Charge (m/z)

9e

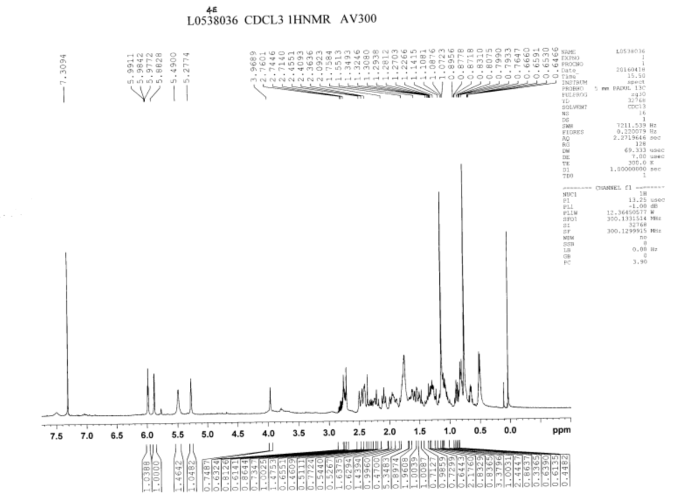
4-E
L0538036 CDCL3 1HNMR AV300
ppm

L0538036 C13-NMR CDCl3 303K AV-300

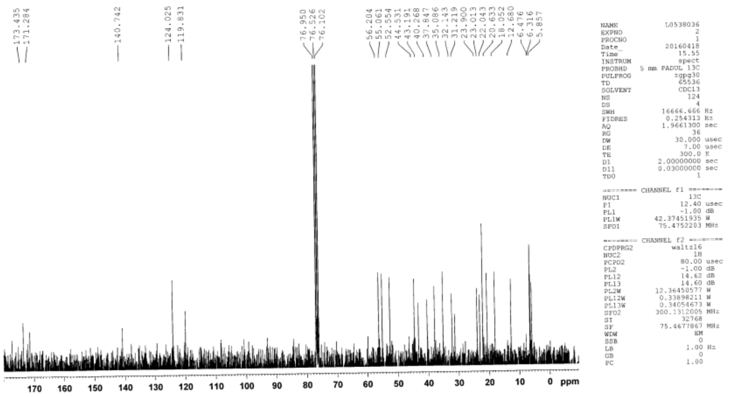

**Sample Name**		**Position**	P2D7	**Instrument Name**	Instrument 1
**User Name**		**Inj Vol**	0.05	**InjPosition**	
**Sample Type**	Sample	**IRM Calibration Status**	Success	**Data Filename**	5C-p.d
**ACQ Method**	20110418-MSonly-p.m	**Comment**		**Acquired Time**	5/9/2017 2:42:44 PM (UTC+08:00)

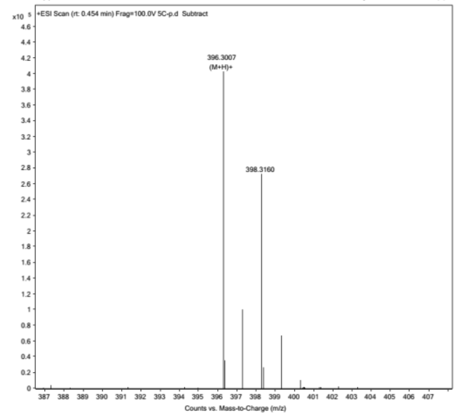

Supplement: Supplemental Material [file IENZ_A_1654469_SM5909.pdf]
